# Progress of Health Plans Toward Meeting the Million Hearts Clinical Target for High Blood Pressure Control — United States, 2010–2012

**Published:** 2014-02-14

**Authors:** Milesh M. Patel, Bennett Datu, Dan Roman, Mary B. Barton, Matthew D. Ritchey, Hilary K. Wall, Fleetwood Loustalot

**Affiliations:** 1 National Committee for Quality Assurance; 2 Division for Heart Disease and Stroke Prevention, National Center for Chronic Disease Prevention and Health Promotion, CDC

High blood pressure is a major cardiovascular disease risk factor and contributed to >362,895 deaths in the United States during 2010 (1). Approximately 67 million persons in the United States have high blood pressure, and only half of those have their condition under control (2). An estimated 46,000 deaths could be avoided annually if 70% of patients with high blood pressure were treated according to published guidelines (3,4). To assess blood pressure control among persons with health insurance, CDC and the National Committee for Quality Assurance (NCQA) examined data in the 2010–2012 Healthcare Effectiveness Data and Information Set (HEDIS). In 2012, approximately 113 million adults aged 18–85 years were covered by health plans measured by HEDIS. The HEDIS controlling blood pressure (CBP) performance measure is the proportion of enrollees with a diagnosis of high blood pressure confirmed in their medical record whose blood pressure is controlled. Overall, only 64% of enrollees with diagnosed high blood pressure in HEDIS-reporting plans had documentation that their blood pressure was controlled. Although these findings signal that additional work is needed to meet the 70% target, modest improvements since 2010, coupled with focused efforts, might make it achievable.

NCQA developed HEDIS to measure the performance in care and service of health insurance plans. HEDIS measures are reported by two thirds of all U.S. health plans, representing approximately three fourths of the U.S. population receiving managed care. To account for differences in population demographics and coverage, NCQA usually collects and reports HEDIS results by Medicare, Medicaid, and commercial health plan categories. Because of differences in how health maintenance organizations (HMOs) and preferred provider organizations (PPOs) capture some data, NCQA further stratifies results by reporting plan type. This report provides aggregate national and adjusted regional estimates and rates reported by plan category and type.*

All plans that reported enrollment figures and valid CBP HEDIS measure rates† were included in the calculation of the percentage of patients seen with diagnosed hypertension.§ NCQA defines a patient with hypertension as a plan member, aged 18–85 years, who had one or more outpatient encounters in which a diagnosis of hypertension that was not pregnancy-related or complicated by end-stage renal disease was recorded¶ during the first 6 months of the measurement period. The CBP measure denominator is calculated by systematically drawing a sample of members who met the definition and had further confirmation of their hypertension diagnosis in the medical record.** The numerator is the population in the denominator who demonstrated blood pressure control (i.e., systolic pressure <140 mmHg and diastolic pressure <90 mmHg).†† Results are expressed in the context of CBP measure values for health plans 1) representing the 50th (i.e., median value) and 90th (i.e., top 10% of performing plans) percentiles for the measure, and 2) meeting the 70% control rate, with additional stratification by NCQA accreditation status.§§ Binary logistic regression was used to estimate region and accreditation status effects on the proportion of plans meeting the 70% control rate while adjusting for plan category/type and reporting year. The significance (−2 log likelihood statistic) and fit of the resulting logistic regression model (area under the curve and Hosmer-Lemeshow Goodness of Fit test) was evaluated.

In 2012, approximately 113.4 million members were covered under plans that reported valid CBP rates (Table 1). Nationally, nearly 11% of members (approximately 12.4 million) had confirmed hypertension and were eligible for the CBP measure; of those, 64% (7.9 million) had their high blood pressure under control. Adjusted control rates were ≥60% for all U.S. Department of Health and Human Services (HHS) regions,¶¶ with rates of 59.5%–68.2% across regions.

Modest improvements occurred in the 50th and 90th percentile plan-level rates from 2010 to 2012 (Table 2). In 2012, 50th percentile rates for all plan categories/types were below the clinical target of 70%, and 90th percentile rates were ≥70% for only commercial and Medicare HMOs and Medicare PPOs. Adjusted odds ratios for meeting the 70% target rate demonstrated that performance improved over time, with differences between regions and plan categories/types; NCQA-accredited plans had greater success than nonaccredited plans (Table 3).

## Editorial Note

In 2012, HHS launched the Million Hearts initiative.*** For clinical settings, one of the Million Hearts goals is to achieve ≥70% control among U.S. adults with diagnosed hypertension by 2017. Overall, HEDIS-reporting plans were 72% more likely to have CBP measure rates meeting this target in 2012 than in 2010. However, despite these improvements, the median rates for the measure among all plan categories/types in 2012 was below this target, and the top 10% of performing plans were barely achieving it. In particular, <15% of Medicare and commercial PPOs met the target. Commercial and Medicare HMOs were twice as likely to have met the target, but <30% were successful. NCQA-accredited plans were twice as likely to have met the 70% clinical target as nonaccredited programs, with the highest percentages occurring among accredited commercial and Medicare Advantage HMOs. The extra level of accountability taken on by accredited plans might better focus their efforts on improving blood pressure control for their members with hypertension.

The percent of patients seen with diagnosed hypertension was greatest in the southeastern states associated with the “stroke belt” (HHS regions 3, 4, and 6), a geographically identified region of high stroke morbidity and mortality (5). Blood pressure control was worst in the Northwest and South (HHS regions 4, 6, and 10). HHS region 10, in the Northwest, has low antihypertensive medication use among persons with self-reported hypertension (6). In the South, despite higher antihypertensive medication use (6), overall blood pressure control is worse than in most other regions. Blacks represent a larger proportion of the population in this region compared with others (7), and despite being more aware of and likely to be treated for their hypertension than whites, blacks are less likely to have their high blood pressure controlled (8).

What is already known on this topic?

Uncontrolled high blood pressure is a major public health problem. Focused efforts to improve blood pressure control can greatly improve health outcomes. Performance measures can be used to assess the effectiveness of health insurance plans in controlling high blood pressure among their members with hypertension.

What is added by this report?

In 2012, nearly 113.4 million members were covered under plans that reported valid Healthcare Effectiveness Data and Information Set (HEDIS) controlling high blood pressure (CBP) performance rates. Nationally, nearly 11% of plan members were eligible for the CBP measure, of whom 64% had their blood pressure under control. Adjusted control rates were ≥60% (range = 59.5%–68.2%) for all U.S. Health and Human Services regions, which was a modest improvement from 2010 rates.

What are the implications for public health practice?

Based on recent improvements measured through HEDIS, the Million Hearts clinical target of ≥70% blood pressure control among hypertensive patients by 2017 is achievable, but further work is needed to effectively identify, monitor, and treat patients with hypertension.

The findings in this report are subject to at least five limitations. First, HEDIS data are limited to those persons insured by reporting health plans. This excludes all fee-for-service Medicare members, a group with a considerable hypertension burden. Second, the CBP measure is based on a sample of plan members with diagnosed hypertension treated during the first 6 months of each reporting year; therefore, the reported percentage of patients seen with diagnosed hypertension should not be misconstrued as a prevalence estimate, because hypertension prevalence among all U.S. adults aged ≥18 years is approximately 30% (2). Third, the CBP measure does not capture persons who have hypertension, but have no recorded diagnosis in the medical record; therefore, it does not describe the effectiveness of plans in identifying hypertension among its members, but only the control of blood pressure among those with documented hypertension diagnoses. Control rates might be overestimated if the proportion of members with undiagnosed hypertension is high. Fourth, it was impossible to risk-adjust HEDIS results to account for population differences (e.g., chronic disease comorbidity prevalence) when comparing CBP values across category/plan types and regions (9). Finally, plans can be attributed to multiple HHS regions because of service area overlap; therefore, some larger plans might be overrepresented across multiple regions, potentially minimizing findings of differences by region.

Performance measures such as HEDIS are tools that can be used to promote health initiatives and assess their effectiveness. They can be used to recognize successful plans and identify areas for improvement (10). Additionally, public reporting on these measures and including the results in accreditation might spur providers and the plans they work with to follow evidence-based treatment guidelines and effectively track management of their hypertensive patients. Million Hearts encourages health plans to continue improvements in the identification, monitoring, and treatment of patients with hypertension. Strategies for improvement might include supporting the implementation of standardized hypertension treatment protocols and health information technology in clinical settings and modifications in health-care coverage/reimbursement (e.g., improved coverage of clinical preventive services and reduced medication copayments).

## Figures and Tables

**TABLE 1:** Blood pressure control among health plan members with diagnosed hypertension,* by plan category, type, and U.S. Department of Health and Human Services (HHS) region† — Healthcare Effectiveness Data and Information Set (HEDIS), 2012

Region§	HEDIS reporting and membership		Patients with diagnosed hypertension			Hypertensive patients with controlled blood pressure		
		
	Plans	Members (millions)	No. (millions)	Members (%)		No. (millions)	Controlled (%)	
	
				Raw	Adjusted§		Raw	Adjusted¶
**National**	**894**	**113.44**	**12.36**	**(10.9)**	**—**	**7.91**	**(64.0)**	**—**
Commercial HMO	193	34.54	2.94	(8.5)	—	2.03	(69.2)	—
Commercial PPO	140	53.70	4.36	(8.1)	—	2.57	(58.8)	—
Medicaid	119	13.82	0.45	(3.3)	—	0.26	(57.0)	—
Medicare HMO	310	8.16	3.30	(40.5)	—	2.25	(68.1)	—
Medicare PPO	132	3.22	1.30	(40.5)	—	0.80	(61.2)	—
**HHS Region (Headquarters)**								
1 (Boston)	82	7.52	0.76	(10.1)	(10.7)	0.51	(66.9)	(65.9)
2 (New York)	108	14.73	1.74	(11.8)	(11.4)	1.10	(63.2)	(62.7)
3 (Philadelphia)	123	13.10	1.72	(13.1)	(12.2)	1.09	(63.6)	(63.0)
4 (Atlanta)	164	21.05	2.86	(13.6)	(12.6)	1.69	(59.0)	(59.5)
5 (Chicago)	188	18.49	2.20	(11.9)	(10.9)	1.42	(64.5)	(65.0)
6 (Dallas)	99	9.74	1.31	(13.4)	(11.4)	0.78	(59.7)	(59.5)
7 (Kansas City)	77	4.83	0.75	(15.5)	(10.8)**	0.48	(63.6)	(64.8)
8 (Denver)	44	3.43	0.29	(8.4)	(7.3)	0.19	(67.5)	(67.6)
9 (San Francisco)	114	23.38	2.55	(10.9)	(10.0)	1.78	(69.8)	(68.2)
10 (Seattle)	66	5.15	0.49	(9.5)	(8.0)	0.30	(61.0)	(60.3)

**Abbreviations:** HMO = health maintenance organization; PPO = preferred provider organization.

* The percentage of patients seen with diagnosed hypertension is not a measure of hypertension prevalence, but describes the number of patients with disease meeting the hypertension case definition that were seen during the first 6 months of the calendar year divided by the total number of health plan beneficiaries aged 18–85 years.

† Listed with headquarters city for each region; territories not included. *Region 1* (Boston): Connecticut, Maine, Maryland, New Hampshire, Rhode Island, and Vermont; *Region 2* (New York): New Jersey and New York; *Region 3* (Philadelphia): Delaware, District of Columbia, Maryland, Pennsylvania, Virginia, and West Virginia; *Region 4* (Atlanta): Alabama, Georgia, Kentucky, Mississippi, North Carolina, South Carolina, and Tennessee; *Region 5* (Chicago): Illinois, Indiana, Michigan, Minnesota, Ohio, and Wisconsin; *Region 6* (Dallas): Arkansas, Louisiana, New Mexico, Oklahoma, and Texas; *Region 7* (Kansas City): Iowa, Kansas, Missouri, and Nebraska; *Region 8* (Denver): Colorado, Montana, North Dakota, South Dakota, Utah, and Wyoming; *Region 9* (San Francisco): Arizona, California, Hawaii and Nevada; *Region 10* (Seattle): Alaska, Idaho, Oregon, and Washington.

§ Individual plans can be associated with multiple HHS regions. Within a given region, all plans associated with that region will contribute to the results for that region. Therefore, regional counts will not necessarily add up to the national counts.

¶ Regional values were adjusted to account for differences in plan distribution across HHS regions. The reference population was the overall number of members, aged 18–85 years, in each reporting health plan category and type.

** The proportion of members covered under Medicaid plans in HHS Region 7 was nearly double that of other regions, explaining why its adjusted rate is much lower than its unadjusted rate.

**TABLE 2:** Proportion of members with diagnosed hypertension with controlled blood pressure by health plan performance and percentage of health plans meeting the ≥70% blood pressure control target, by health plan category, type, and year—Healthcare Effectiveness Data and Information Set, 2010–2012

Plan category	Reporting plan type	Year	Plans	Hypertensive plan members with controlled blood pressure, by plan performance percentile (%)*		Plans that met the target of ≥70% blood pressure control among plan members with diagnosed hypertension (%)		
	
				50th	90th	Overall	Nonaccredited	Accredited
Commercial	HMO	2010	238	(65.0)	(73.0)	(23.1)	(14.9)	(25.1)
		2011	218	(65.2)	(74.1)	(21.6)	(9.6)	(25.3)
		2012	199	(66.3)	(76.2)	(28.6)	(14.0)	(32.7)
	PPO	2010	40	(49.9)	(64.8)	(5.0)	(0.0)	(16.7)
		2011	96	(56.3)	(67.6)	(5.2)	(5.6)	(5.0)
		2012	141	(59.9)	(68.2)	(7.1)	(5.0)	(7.4)
Medicaid	HMO	2010	128	(57.1)	(67.2)	(5.5)	(3.3)	(7.4)
		2011	137	(56.4)	(67.6)	(4.4)	(3.1)	(5.5)
		2012	148	(57.5)	(69.1)	(8.1)	(5.2)	(10.0)
Medicare Advantage	HMO	2010	289	(62.3)	(71.6)	(14.9)	(9.4)	(25.5)
		2011	309	(63.4)	(74.4)	(22.7)	(16.9)	(32.5)
		2012	310	(64.4)	(75.5)	(26.8)	(21.0)	(35.5)
	PPO	2010	87	(55.5)	(67.2)	(5.8)	(7.2)	(0.0)
		2011	123	(55.0)	(69.0)	(8.9)	(5.3)	(21.4)
		2012	132	(60.7)	(70.9)	(14.4)	(15.6)	(11.9)

**Abbreviations:** HMO = health maintenance organization; PPO = preferred provider organization.

* The controlling blood pressure (CBP) measure value of health plans at the 50th and 90th percentiles for the measure. Fifty percent of health plans had better (i.e., higher) CBP measure values than the health plan that represents the 50th percentile and 10% of plans had better values than the health plan that represents the 90th percentile.

**TABLE 3:** Adjusted odds ratios for meeting the target for blood pressure control of ≥70% among health plan members with diagnosed hypertension — Healthcare Effectiveness Data and Information Set, 2010–2012

Characteristic	Comparison	Odds ratio	(95% CI)
Plan category	Medicaid versus commercial	0.21	(0.14–0.34)
	Medicare Advantage versus commercial	1.44	(1.11–1.86)
Reporting plan type	PPO versus HMO	0.30	(0.22–0.42)
Reporting year	2012 versus 2010	1.72	(1.30–2.27)
	2012 versus 2011	1.37	(1.05–1.79)
Accreditation status	“Yes” versus “no”	2.00	(1.55–2.58)
HHS Region (Headquarters)*	1 (Boston) versus others	1.76	(1.12–2.77)
	2 (New York) versus others	1.03†	(0.67–1.59)†
	3 (Philadelphia) versus others	1.26†	(0.83–1.91)†
	4 (Atlanta) versus others	0.24	(0.15–0.40)
	5 (Chicago) versus others	1.49	(1.02–2.18)
	6 (Dallas) versus others	0.12	(0.05–0.27)
	7 (Kansas City) versus others	0.63†	(0.38–1.03)†
	8 (Denver) versus others	1.32†	(0.76–2.31)†
	9 (San Francisco) versus others	1.04†	(0.66–1.63)†
	10 (Seattle) versus others	0.32	(0.16–0.63)

**Abbreviations:** CI = confidence interval; HHS = U.S. Department of Health and Human Services; HMO = health maintenance organization; PPO = preferred provider organization.

* Listed with headquarters city for each region; territories not included. *Region 1* (Boston): Connecticut, Maine, Maryland, New Hampshire, Rhode Island, and Vermont; *Region 2* (New York): New Jersey and New York; *Region 3* (Philadelphia): Delaware, District of Columbia, Maryland, Pennsylvania, Virginia, and West Virginia; *Region 4* (Atlanta): Alabama, Georgia, Kentucky, Mississippi, North Carolina, South Carolina, and Tennessee; *Region 5* (Chicago): Illinois, Indiana, Michigan, Minnesota, Ohio, and Wisconsin; *Region 6* (Dallas): Arkansas, Louisiana, New Mexico, Oklahoma, and Texas; *Region 7* (Kansas City): Iowa, Kansas, Missouri, and Nebraska; *Region 8* (Denver): Colorado, Montana, North Dakota, South Dakota, Utah, and Wyoming; *Region 9* (San Francisco): Arizona, California, Hawaii and Nevada; *Region 10* (Seattle): Alaska, Idaho, Oregon, and Washington.

† Denotes no statistically significant association (p≥0.05).

## Footnotes

* Regional values are adjusted to account for differences in plan distribution across HHS regions. The reference population was the overall number of members, aged 18–85 years, in each reporting health plan category and type in 2010. Before 2010, fewer than five PPOs in each category reported valid CBP measures.

† Defined as having ≥30 patients in the target population sample (CBP measure denominator) and passing the NCQA audit review.

§ The percentage of patients seen with diagnosed hypertension is not a measure of hypertension prevalence, but describes the number of patients with diagnosed hypertension who were seen during the first 6 months of the calendar year divided by the total number of health plan members aged 18–85 years.

¶ *International Classification of Diseases, Ninth Revision, Clinical Modification* code of 401.

** To confirm the diagnosis of hypertension (HTN), the organization must find notation of one of the following in the medical record on or before June 30 of the measurement year: HTN, high blood pressure, elevated blood pressure, borderline HTN, intermittent HTN, history of HTN, hypertensive vascular disease, hyperpiesia, or hyperpiesis.

†† Based on their most recent blood pressure readings. If multiple blood pressure measurements occurred on the same date, or were noted in the medical record on the same date, the lowest systolic and lowest diastolic blood pressure readings were used.

§§ NCQA health plan accreditation includes two major components on which a plan’s performance is scored: 1) standards—an evaluation of the plan’s structure and processes to maintain and improve quality in five core areas, and 2) HEDIS—an evaluation of the plan’s performance on process and outcomes in clinical care and member experience of care. A health plan is considered to be NCQA-accredited if it achieved “excellent,” “commendable,” or “accredited” status for the performance year. Additional information is available at http://www.ncqa.org/programs/accreditation.aspx.

¶¶ The HHS regions, listed with headquarters city for each, territories not included, are as follows: *Region 1* (Boston): Connecticut, Maine, Maryland, New Hampshire, Rhode Island, and Vermont; *Region 2* (New York): New Jersey and New York; *Region 3* (Philadelphia): Delaware, District of Columbia, Maryland, Pennsylvania, Virginia, and West Virginia; *Region 4* (Atlanta): Alabama, Georgia, Kentucky, Mississippi, North Carolina, South Carolina, and Tennessee; *Region 5* (Chicago): Illinois, Indiana, Michigan, Minnesota, Ohio, and Wisconsin; *Region 6* (Dallas): Arkansas, Louisiana, New Mexico, Oklahoma, and Texas; *Region 7* (Kansas City): Iowa, Kansas, Missouri, and Nebraska; *Region 8* (Denver): Colorado, Montana, North Dakota, South Dakota, Utah, and Wyoming; *Region 9* (San Francisco): Arizona, California, Hawaii and Nevada; *Region 10* (Seattle): Alaska, Idaho, Oregon, and Washington.

*** HHS, in collaboration with nonprofit and private organizations, launched Million Hearts ( http://www.millionhearts.hhs.gov ), a combination of clinical and community evidence-based interventions and strategies aimed at preventing 1 million heart attacks and strokes during the 5-year period of 2012–2016.
